# The Association Between Play Fighting and Information Gathering during Subsequent Contests

**DOI:** 10.1038/s41598-020-58063-x

**Published:** 2020-01-24

**Authors:** Jennifer E. Weller, Simon P. Turner, Marianne Farish, Irene Camerlink, Gareth Arnott

**Affiliations:** 1 Institute for Global Food Security, School of Biological Sciences, Queens University Belfast, Belfast, UK; 2 Animal Behaviour & Welfare, Scotland’s Rural College (SRUC), Edinburgh, UK; 3 Institute of Animal Welfare Science, University of Veterinary Medicine Vienna (Vetmeduni), Vienna, Austria

**Keywords:** Evolution, Zoology

## Abstract

Many hypotheses regarding the evolution of social play have been suggested, including the development of later-life assessment skills. However, the link between play fighting experience and information gathering during contests has yet to be examined. This paper explores the association between play fighting and contest assessment strategy in the domestic pig (*Sus scrofa*). Using an established framework, we provide evidence suggesting play fighting frequency may affect the extent to which individuals incorporate information regarding their own and their competitors’ resource holding potential (RHP) in escalation decisions. Pigs were allocated as ‘high play’ or ‘low play’ based upon their relative play fighting frequency. To maximise variation in play, 12 litters underwent a socialisation treatment while the remaining 12 litters were kept isolated within their home pen (i.e. control treatment). At eight weeks of age contests were staged between pairs of unfamiliar pigs, using 19 ‘high play’ dyads and 19 ‘low play’ dyads. While ‘high play’ dyads were observed to rely on a pure self-assessment strategy, ‘low play dyads’ did not meet the predictions of either self- or mutual assessment, suggesting their contest behaviour may have been motivated by alternative factors. We suggest that early life play fighting may therefore allow individuals to develop an accurate estimate of their RHP.

## Introduction

The evolutionary function of play has long been debated within the study of animal behaviour^1–4^. However, despite lacking a formal definition (beyond the five criteria provided by Burghardt^3^), the taxonomic prevalence and evolutionary persistence of play can be attributed to one basic principle: the cost of its performance must be outweighed by the fitness benefit received by the performer^1^. As play is considered to be costly in terms of both time, resources, and increased risk of predation^5,6^ many short-and long-term benefits have been proposed, resulting in the formation of three key hypothesis regarding its evolutionary purpose.

Firstly, the motor training hypothesis predicts that juvenile activity such as play allows for the improvement of motor performance through the development of the brain and peripheral nervous system, muscles, bones and connective tissue, and/or the cardiovascular system^7^. Initially it was suggested that play aids in the growth and retention of rarely used muscles that are critical for the performance of certain adult activities, such as fighting or escape^8^. However more recent investigation into its temporal overlap with sensitive early life developmental periods suggests that play may instead facilitate the differentiation of skeletal muscle fibres and cerebellar synaptogenesis^9^. Secondly, the ‘training for the unexpected’ hypothesis^2^, suggests that animals actively seek situations in which they experience a sudden loss of control though a means of self-handicapping. As such, individuals are predicted to develop both the appropriate emotional and physical response to unexpected events when the cost of mistakes is low.

Thirdly, early life play may aid in social cohesion^10,11^ and the development of social skills or behaviours required for later life interactions^5,12^. While juvenile play has been suggested to aid in the formation of strong social bonds^5,13^ that are required for cooperation, it may also serve to ameliorate tensions between competing individuals within a social group^11,14–16^. Additionally, given the similarities observed between juvenile social play and certain adult behaviours (such as fighting and mating), it may provide individuals with an opportunity to practice and develop the skills required to perform these behaviours in a relatively safe environment^17,18^, where social transgressions are quickly forgiven^12^.

For example, play fighting as a juvenile has often been assumed to relate to fighting ability later in life^19–23^. However, direct testing of this hypothesis has been limited and the results yielded have often been contradictory^24^. While Sharpe^25^ observed no effect of juvenile social play on contest success in meerkats (*Suricata suricatta*), a recent study in the domestic pig (*Sus scrofa*) found evidence to suggest that pre-weaning play fighting frequency increased contest success in females, and decreased it in males^26^. Furthermore, the dominance ranks calculated for juvenile and yearling yellow-bellied marmots using the directional outcomes of their playful interactions were found to correlate with their later life dominance ranks as calculated from agonistic encounters^27^.

Another potential benefit of early life play that has yet to be explored is the refinement of social assessment skills^5,17^. Croft and Snaith^28^ proposed that playful sparring allowed red kangaroos (*Macropus rufus*) to gather information about their own fighting ability relative to that of the general population at a relatively low cost. Here we expand upon this hypothesis by suggesting that play may improve the skill with which individuals can accurately accrue information regarding their opponent’s fighting ability and help refine the use of such information during contests.

Given that contests are typically costly to all competitors^29–32^ selection should favour individuals that are able to make tactical decisions regarding the escalation of aggressive behaviour^31,33–35^. These decisions are likely to depend highly on two contributing factors; the benefit/cost of winning/losing the encounter (i.e. gained/lost resource value), and the fighting ability of the competing individuals^35–37^. While it is generally accepted than animals will adjust their fighting strategy based upon perceptions of resource value^33,36–38^, the extent to which an individual can gather and act upon information regarding its own, and/or its opponent’s fighting ability (commonly termed resource holding potential or RHP^33^), is still debated^39,40^. Multiple strategies of contest assessment have been proposed, which can be separated into two main categories based on their fundamental assumptions^41^.

According to self-assessment models (e.g. ‘War of attrition without assessment’^42^; ‘Energetic war of attrition’^43,44^; ‘Cumulative assessment model’ (CAM)^45^), individuals are unable to obtain information regarding their opponent’s RHP and therefore continue to escalate contests until a maximum cost threshold is reached. This threshold is dictated solely by the individual’s RHP and the value they place upon the disputed resource. With the exception of CAM (which predicts that individuals are able to inflict additional costs upon their opponent) these models assume that costs are purely accrued from the actions of the performer. Subsequently, contests continue until the weaker individual reaches their maximum threshold and retreats.

Mutual assessment models on the other hand (e.g. ‘Asymmetric war of attrition’^35,46^; ‘Sequential assessment model’ (SAM)^47,48^) assume that contestants are able to gather information about their competitor’s RHP and subsequently evaluate their own RHP relative to that of the opponent’s. This allows individuals to predict the outcome of escalated conflict and make informed decisions regarding continued escalation. As such, the individual with the lowest RHP is able to terminate a contest they are unlikely to win before further costs are accrued.

**Figure 1:**
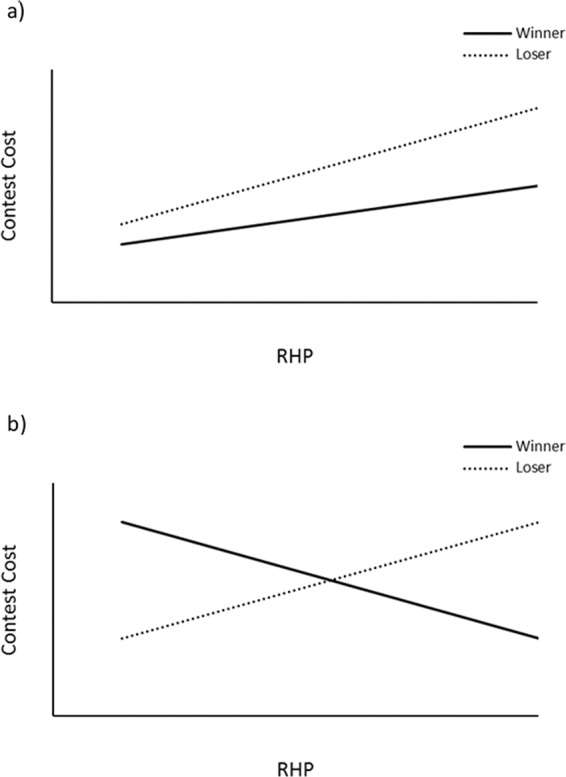
Relationships predicted between the resource-holding potential of both winning and losing individuals and contest cost for alternative assessment hypothesis as suggested by Taylor & Elwood (2003). (**a**) Pure self-assessment. (**b**) Mutual assessment or Cumulative Assessment (CAM).

In this study we use an established framework^41,49^ to investigate the association between early life play frequency and subsequent contest assessment ability in the domestic pig (*Sus scrofa*). Prior to the development of this framework^41^, researchers relied on finding a negative relationship between RHP difference and contest cost (typically duration), which was said to be “diagnostic” of mutual assessment. However, Taylor and Elwood^41^ clearly demonstrated that such a negative relationship can also occur with self-assessment. To avoid spurious conclusions, they developed and advocated a framework that involves examining associations between individual contestant RHP and contest cost separately for winners and losers. Here, we consider the extent to which winner and loser RHP (represented here by body weight) influences the cost of contests staged between two individuals that experienced high levels of pre-weaning play fighting and two individuals that experienced low levels of pre-weaning play fighting. Under self-assessment, contest cost is predicted to be positively correlated with both loser, and to a lesser extent, winner RHP. On the other hand, under mutual assessment (and CAM), contest duration is predicted to correlate positively with loser RHP and negatively with winner RHP (Fig. 1).

Body weight was chosen as a correlated measure of individual RHP as animal contests are typically won by the larger or heavier opponent^36,50,51^. Additionally, the use of body weight as a tool for considering assessment strategies has been widely adopted^49,52,53^. It is predicted that individuals with high levels of pre-weaning play experience will be better able to assess their opponent’s RHP and will subsequently adopt a mutual assessment strategy when engaging in contest behaviour. Additionally, high play individuals are predicted to demonstrate a more accurate understanding of their own RHP, having experienced more opportunities to compare their own fighting ability relative to that of their play partners.

Due to previous concerns regarding the validity of contest duration and mutual fighting duration as measures of contest cost^49,54–56^ an additional six physical and physiological variables that more directly measure contest costs were also collected; winner skin lesions, loser skin lesions, proportional increase in winner blood glucose (hereafter Δ winner blood glucose), proportional increase in loser blood glucose (hereafter Δ loser blood glucose), proportional increase in winner blood lactate (hereafter Δ winner blood lactate), and proportional increase in loser blood lactate (hereafter Δ loser blood lactate). These measures of contest cost were then reduced to a single factor using principal component analysis (PCA^57^). PCA has been widely used across many areas of animal behaviour^58–62^, allowing multiple measures to be reduced into more manageable principal components. However, to our knowledge this is the first study to explore contest assessment using this approach (i.e. the use of a composite measure of overall contest cost to examine assessment strategy).

## Methods

### Ethical note

All procedures were performed in accordance with the UK Government Defra animal welfare codes and followed the recommended European Guidelines for accommodation and care of animals as well as the ASAB/ABS guidelines for the treatment of animals in behavioural research and teaching. Work was approved by both the UK Government Home Office (Project licence PPL60/4330) under the Animals Scientific Procedures Act of 1986 and SRUC’s Animal ethics committee (no. ED RP 04-2014). All stages of the experiment were conducted in liaison with a licenced SRUC veterinary surgeon.

### Animals and housing

A total of 263 piglets (138 males and 125 females) from 24 litters were used over the course of this study. Piglets were born to 24 Large White × Landrace sows served by American Hampshire boars in 2 batches housed between January and February 2016. Each sow only contributed one litter to the study. Piglets were only fostered when necessary for survival. Teeth and tails were kept intact and males were not castrated. Piglets were born and raised in conventional farrowing pens located at the SRUC Easter Howgate pig unit (Roslin, Scotland). Throughout the pre-weaning period sows remained confined in traditional farrowing crates while their piglets were able to move freely around the surrounding 2.50 m × 1.50 m pen. Piglets also had access to an additional 0.5 m × 1.50 m heated creep area located in front of the sow’s crate via two entrances. Water was available from drinkers *ab libitum* and starter creep feed was provided to the piglets from approximately 21 days of age.

In order to ensure the widest possible range of play fighting experience, 12 litters (6 litters from each batch) underwent a socialisation process in which piglets were able to access a neighbouring pen and interact with non-littermates between 14 days post-partum and weaning on day 28. This was achieved by the installation of a pen partition containing a ~35 × 74 cm opening located between the middle and rear of the sow. The remaining 12 litters were kept isolated within their home pens in order to act as controls. Although previous work regarding the pre-weaning socialisation of piglets has found no evidence to suggest socialisation influenced the frequency of early life play experience, socialised individuals directed approximately one third of their piglet directed play (including play fighting) towards non-littermates^56^. Weaning was performed at day 28 by removal of the sow from the farrowing crate, which is a standard practice at Easter Howgate pig unit. Piglets were then weighed, ear tagged for individual recognition, vaccinated, and relocated to testing pens measuring 1.9 × 5.8 m. Socialised litters were once again separated and pigs were housed in their original litter groups. Pens were cleaned and provided with fresh straw daily while water and commercial feed was available *ad libitum*. Once pigs had acclimatised to the new pens, they were gradually habituated to human contact and being moved between pens. Using diminishing groups sizes, pigs where habituated to the test holding pens until they were able to remain in the holding pens alone for a minimum of three minutes.

### Measuring play

To determine the pre-weaning play fighting experience of piglets, Geovision surveillance cameras linked to GV-1480 playback software were installed above all 12 farrowing pens. No video footage was available from inside the heated creep. Piglet play behaviour was recorded between 10:00 and 16:00 on days 14, 16, 19, 21, 24, and 26 and played back using EZViewLog500. The number of play fighting bouts performed was coded for the first 15 minutes of every hour using a clearly defined ethogram (see Supplementary Table S1). All piglets were observed simultaneously and all observations were made by a single observer. Videos were paused and rewound if multiple interactions occurred at once. From this, the total play fighting frequency (the sum of all accepted play invites received and all successful invites made) of all socialised and control piglets was calculated.

### Dyadic contests

A total of 76 dyads containing two unfamiliar individuals from the same treatment group (Socialised: 46, Control: 30) were randomly formed at 8 weeks of age. Contests were performed in a neutral, novel, testing arena (2.9 × 3.8 m) that allowed opponents to enter from two separate holding pens via two opposite-facing gates. Contests were considered to have begun once both pigs entered the arena and were ended when either individual (hereafter referred to as the loser) performed a successful retreat (i.e. no retaliation attempt made within one minute of the retreat being performed). If no clear winner could be identified within 20 minutes of pigs entering the pen the contest was ended by the researchers. Contests were also ended immediately if fighting was deemed to be too severe (as determined by skin lesions and risk of lameness), if either individual displayed repeated fear behaviours (such as sustained vocalization or escape attempts), or if one pig was repeatedly mounted. Food was not present during the contest as pigs typically engage in contest behaviour upon initial introduction to a non-familiar conspecific in order to establish a dominance hierarchy even in the absence of resources^26,49,55^. It is this hierarchy that later established priority of access to tangible resources.

### Measuring contest costs

In addition to contest duration, the duration of mutual fighting behaviour (defined as an aggressive act made by either pig directed towards their opponent resulting in a retaliatory aggressive act occurring within 5 seconds^26^) was also recorded. Contests were recorded using a Canon Legria HFS21 camcorder set at standard definition with a wide-angle lens and positioned 5 m above the contest arena at a 30 degree angle. In order to calculate the duration of mutual fighting videos were played back in Noldus Observer XT10. Furthermore, skin lesions (defined as scratches located anywhere on the body resulting from being bitten) were counted for both individuals immediately before and after the contest, allowing the number of skin lesions accrued during the contest to be calculated.

In order to quantify physiological measures of contest cost blood glucose and lactate were assayed, to reflect the energetics of aggression^55^, by collecting a small drop of blood from both competitors pre-and post-contest. Prior to the performance of contests, pigs were habituated to being handled in the sampling crate and having their ears touched over the course of two weeks. Immediately before and after a contest, blood samples were collected from the ear vein of competing pigs using a flat-bladed capillary blood lancet and then applied directly to the test strips of both a blood glucose (iDia Blood Glucose Meter) and blood lactate (The EDGE Lactate Analyser) meter. Due to the range limitation of the lactate meter (0.7–22.2 mmol/litre) two individuals reported a blood lactate concentration of below the minimum threshold pre-contest and were therefore given a reading of 0.7 mmol/litres. Furthermore, one individual measured above the limitation threshold post-contest and was given a reading of 22.2 mmol/litre. Δ blood glucose and Δ blood lactate was then calculated for both the winner and loser of the contest.

### Statistical analysis

All data analysis was performed using the statistical package R version 3.4.0 (The R Foundation for Statistical Computing) and SPSS Statistics (IBM Version 26). Data are presented as the mean ± the standard error of the mean. Pre-weaning play fighting behaviour was unavailable for one control group due to technical difficulties. Subsequently, dyads containing these individuals were removed from the analysis. Additionally, 3 dyads failed to present a clear winner within the 20 min time limit while 4 contests were ended by researchers before an outcome could be determined. This resulted in a total of 65 usable dyads with clear contest outcomes.

To determine if an individual experienced a ‘high’ or ‘low’ pre-weaning play fighting frequency, the median frequency of observed play fights was calculated for individuals that engaged in a contest with a clear outcome (n = 130, median = 23.5, IQR = 25). All pigs that engaged in more play fights than the median were designated ‘high’ play individuals (mean play fighting frequency = 40.89 ± SE 1.65) and all individuals that performed fewer play fights than the median were designated ‘low’ play individuals (mean play fighting frequency = 12.72 ± SE 0.67). This resulted in data being available for 38 dyads; 19 dyads containing two ‘high’ play individuals (hereafter referred to as high play dyads; Socialised: 13, Control: 6), and 19 dyads containing two ‘low’ play individuals (hereafter referred to as low play dyads; Socialised: 13, Control: 6).

Due to the non-normality of contest cost data, as indicated by Shapiro-Wilks testing, a series of Mann-Whitney U tests were used to investigate the difference in each of the eight measure of contest cost used between high and low play dyads. In order to investigate the assessment strategy being used, Principal component analysis (PCA^57^) was used to condense contest duration, winner skin lesions, loser skin lesions, Δ winner blood glucose, Δ loser blood glucose, Δ winner blood lactate, and Δ loser blood lactate of high and low play dyads into one dependant variable that represented contest cost. As only 32 of the 38 dyads performed mutual fighting, mutual fighting duration was not included in the PCA and was analysed separately. In order to meet the assumptions of PCA, all contributing variables were checked for normality using a Shapiro-Wilk test and transformed accordingly. PCA uses the linear correlations observed between pairs of variables in order to calculate ‘principal components, which can be used to determine variables that co-vary (in either the same or opposite directions) as indicated by their loading score. Loading scores (given for each variable) represents the correlation between the new principal component score given to each data point and an initial variable, thereby representing the importance of that variable to the principal component^62,63^. Here it was observed that all seven variables contributed strongly to the first principal component (hereafter referred to as PC1; see Table 1) which explained 62% of the variance observed in this dataset. The second principal component only explained an additional 12% of the variance observed and was therefore not included in the subsequent analysis. The PC1 score given to each dyad was therefore considered to be a good representation of contest cost.

**Table 1:** Loading of each contributing variable on the first principal component (PC1) extracted using principal component analysis.

	PC1
Contest Duration^a^	0.792
Winner Skin Lesions^b^	0.855
Loser Skin Lesions^b^	0.823
Δ Winner Blood Glucose^a^	0.734
Δ Loser Blood Glucose^a^	0.825
Δ Winner Blood Lactate^a^	0.747
Δ Loser Blood Lactate^a^	0.749

^a^represents variables that were log transformed. ^b^represents variables that were square-root transformed.

The relationship between dyad play fighting frequency pre-weaning and contest assessment was determined using general linear mixed effects models (GLMMs), containing either PC1 or mutual fighting duration as the dependant variable. Therefore, both exploratory models contained winner weight, loser weight, dyad play (high play vs. low play), and treatment (socialisation vs. control) as fixed effects, while batch was included as a random factor. Model residuals were examined for normality and both indicators of contest cost were subsequently log transformed. Initially models contained all relevant variables and interaction effects, which were then removed from the model using a top-down approach in order to identify the best fitting model, as indicated by the Akaike information criterion (AIC score). Models were examined for fit using maximum likelihood (ML), while test statistics were extracted from the best fitting model by means of a Wald’s test using restricted maximum likelihood (REML).

Weight differences between winners and losers from socialised/control and high/low play dyads were explored using a general linear mixed effects model. Individual weight was considered the dependant variable while contest outcome (i.e. winner or loser), dyad play, and treatment were included as fixed effects. Dyad ID nested with batch was included as a random effect in order to account for the non-independence of opponents^64^. Again, the initial model included all relevant variables, which were removed using the same method as before, and the best fitting model was examined. The effect of play frequency (high or low) and sex on weight was further explored using a factorial ANOVA.

## Results

**Figure 2:**
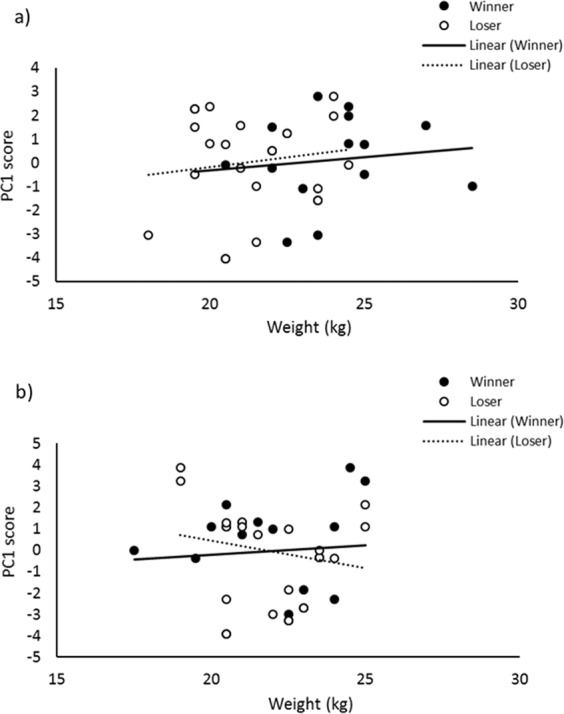
Effect of winner and loser weight (kg) on PC1 score (a composite measure of contest cost) in (**a**) high play dyads and (**b**) low play dyads. Trend lines indicate lines of best fit.

The cost of contests did not differ between high play and low play dyads (Table 2). However, investigation into the effect of pre-weaning play fighting frequency on a composite measure of contest cost (PC1) revealed a three-way interaction effect of winner weight, loser weight, and dyad pre-weaning play fighting frequency (χ^2^_1_ = 6.432, *p* = 0.011; Fig. 2). Within the high play dyads, PC1 was positively related to loser weight (β = 0.161) and, to a lesser extent, winner weight (β = 0.110). On the other hand, in the low play dyads there was a negative relationship between PC1 and loser weight (β = −0.257), while no relationship with winner weight (β = 0.087) was observed. No effect of socialisation or control treatment (socialised or control) was observed on PC1 (χ^2^_2_ = 4.28, *p* = 0.118).When mutual fighting was used as a represented measure of contest cost, no effect of treatment, dyad play frequency, winner weight, or loser weight was observed (*p* > 0.05). All results from GLMMs are presented in Supplementary Table S2.

**Table 2:** Comparison of contest cost between high play and low play dyads. Mean and the standard error of the mean is presented for both treatment groups.

	HH Dyads		LL Dyads		*W*	*n*	*p*
	$\bar{x}$	SE	$\bar{x}$	SE			
Contest Duration (s)	207.84	42.33	278.42	63.87	165	38	0.66
Winner Skin Lesions	38.53	9.17	52.32	12.00	155	38	0.46
Loser Skin Lesions	55.00	10.64	77.79	17.85	147.5	38	0.34
Δ Winner Blood Glucose	1.19	0.07	1.16	0.27	168.5	38	0.74
Δ Loser Blood Glucose	1.30	0.09	1.24	0.29	204	38	0.50
Δ Winner Blood Lactate	5.12	0.79	5.09	1.17	192	38	0.75
Δ Loser Blood Lactate	5.03	0.86	5.44	1.25	191	38	0.77
Mutual Fighting Duration (s)	84.92	29.87	112.25	36.86	113	32	0.64

**Figure 3:**
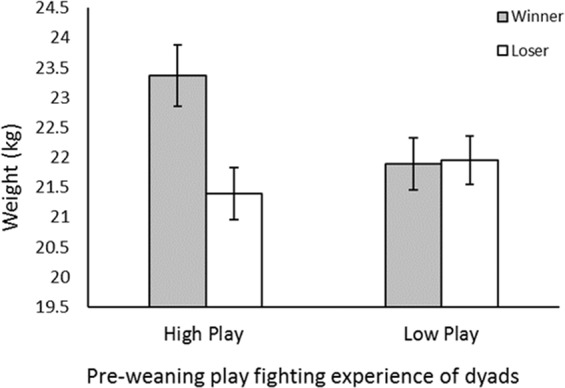
Mean weight (kg) of winning and losing individuals from high pre-weaning play fighting frequency and low pre-weaning play fighting experience dyads at 7 weeks of age. Error bars represent the standard error of the mean.

As expected, an individual’s contest outcome (i.e. winner or loser) was significantly related to their body weight (Winner: 22.63 ± 0.35 kg, Loser: 21.67 ± 0.29 kg; χ^2^_1_ = 4.682, *p* = 0.030). However, a significant interaction effect between outcome and dyad pre-weaning play fighting frequency revealed that while weight was important for determining outcome in the high play dyads, it did not influence contest outcome in the low play dyads (χ^2^_1_ = 5.209, *p* = 0.022; Fig. 3). Neither sex nor play experience was found to influence body weight (Sex: *F*_1,72_ = 0.154, *p* = 0.696; Play: *F*_1,72_ = 0.938, *p* = 0.336).

## Discussion

This study predicted that dyads containing pigs that had experienced a high level of play fighting pre-weaning would be better able to gather information regarding their opponent’s RHP, and make informed decisions regarding the escalation of contests, than dyads containing pigs that had experienced low levels of play fighting. Subsequently, high play dyads were predicted to show evidence of mutual assessment while low play dyads were predicted to rely on either a self- or a no-assessment strategy. Contrary to this, using an established framework^41,49^ this study presents evidence to suggest that high play dyads did not incorporate information regarding their opponent’s RHP into their decisions regarding contest continuation and instead relied on a pure self-assessment strategy during agonistic encounters. In high play dyads, contest cost (represented by a principal component factor explaining 62% of variance) was observed to increase with both loser RHP, and to a lesser extent winner RHP, suggesting that contests continued to escalate until the losing individual reached its maximum cost threshold and subsequently retreated. This is further supported by the finding that in high play dyads contest outcome was influenced by competitor weight; on average, winning individuals from high-play dyads were observed to be significantly heavier than losing individuals.

We suggest that increased early life play frequency may have provided competitors with a prior understanding of their own RHP, as suggested by Thompson^24^, as well as a relative understanding of their RHP compared to that of the general population, as suggested by Croft & Snaith^28^. Subsequently, individuals that experienced high play as juveniles may have been able to base contest decisions solely upon their own maximum cost threshold. While the cognitive ability required to assess an opponent’s RHP is still subject to debate^40,65,66^ it has been acknowledged that individuals are unlikely to have perfect information regarding their own RHP relative to others^67^. It has also been suggested that the outcome of previous contests alters an individual’s perception of its own fighting ability relative to that of the general population^30,68,69^. In the same way, play fighting may provide individuals with an opportunity to make a generalised comparison of their own RHP, all be it in a less costly manner^17,28^. This would be particularly beneficial if the cost of assessing an opponent during a contest is high^30^, or in open social societies where the likelihood of meeting the same individual again is low and substantial investment in assessing an opponent individual’s RHP is unjustified^28^.

Furthermore, having experience of play fighting may serve a similar function to the ‘winner effect’, where individuals that have recently experienced a win are more likely to win their next contest^30,68–70^. While the effect of prior contest outcome on the adoption of assessment strategies has yet to be explored, Camerlink *et al*.^49^ reported that pigs with previous experience of aggressive interactions relied on a pure self-assessment strategy. Pigs with limited prior experience, however, were observed to perform mutual assessment during the pre-escalation stage of contests. This result further highlights a decreased reliance on opponent-directed assessment when prior social knowledge is available, either due to increased confidence in one’s own fighting ability, or an improved understanding of one’s RHP relative to that of the general population.

One the other hand, in this study low play dyads showed a negative relationship between contest cost (as indicated by PC1) and loser RHP. Furthermore, contest cost was observed to be positively related to winner RHP. This contrasts with previous predictions for both self-assessment and mutual assessment^41^ but is consistent with the suggestion that individuals may be more motivated to engage in contests when the size difference between themselves and their opponent is large. While initially it seems non-beneficial for small individuals to show such persistence, several models have been proposed to explain this phenomenon^71–73^.

For example, the ‘desperado effect hypothesis’ proposed by Grafen^71^ suggests that when the fitness benefit of winning a contest is high individuals with a history of losing will start to disregard the asymmetry in RHP between themselves and the opponent. Individuals that repeatedly lose contests have no incentive to respect recognizable asymmetries if they are unable to obtain a vital disputed resource (such as food or mates) through other means. Although the pigs used in this study had no prior experience of losing a post-weaning contest, small individuals may have been more motivated to persist with contests due to external factors, such their social experiences pre-weaning. Furthermore, the ‘Napoleon strategy hypothesis’^72^ suggests that smaller individuals should persist with an agonistic interaction if the resource being disputed is valuable and contest outcome is not solely predicted by RHP.

Here it was observed that weight did not influence contest outcome in low play dyads, suggesting that smaller pigs may have benefited from the continuation of contests regardless of their own RHP. Alternatively, pigs that received low levels of play fighting pre-weaning may have had incorrect information regarding their own RHP. Subsequently, losers from low-play dyads may have based their decision to continue engaging in aggression based upon incorrect information resulting from a lack of pre-weaning play-fighting.

It should also be considered than an additional third factor may have influenced both pre-weaning play fighting frequency and assessment ability. For example, before the emergence of play behaviour, pigs establish a dominance hierarchy with their littermates, as evidenced by teat order^74^, which may influence access to nutrition and growth rate^75^ (but see^76^). Play is a luxury behaviour that is only performed when all other primary needs have been fulfilled^77–79^, and although high play individuals were not observed to be significantly heavier than their low play counterparts, it is possible that these individuals may have obtained better nutrition during the pre-weaning period. Subsequently, these individuals may have been better able to make assessments regarding their own RHP. By dividing pigs based upon play frequency, it is possible that dominant, well-fed pigs were placed in a separate sample than their lower-ranking siblings. Additionally, differences in the sex ratios of the high play and low play dyads may have had an unseen influence on our findings. Further experimentation is required in order to prise apart the influence of play and early life dominance on assessment strategy.

## Conclusion

It has previously been suggested that early life social play allows individuals an opportunity to develop the skills required during the performance of agonistic encounters later in life. While juvenile play fighting is often structurally compared to adult aggression, very few studies have tested this link empirically. Furthermore, the development of non-physical skills, such as assessment ability, have been widely overlooked. This study provides evidence to suggest an association between early life play fighting frequency and the use of assessment strategies in the domestic pig. Pigs classified as having experienced high levels of play fighting were found to base contest escalation decisions purely upon their own RHP, with victory most commonly going to the heavier opponent. On the other hand, low play pigs appeared to adopt a form of assessment in which losing individuals were more motivated to persist with contests if their own RHP was low. This is proposed to result from the ‘desperado effect’, in which smaller individuals place a higher value on contest success and are subsequently prepared to invest higher costs than larger individuals. This highlights the potential impact of juvenile play on adult social behaviour and suggests more focus should be given to early life predictors of contest behaviour.

## Supplementary information

Supporting Information.

## Data Availability

The datasets generated and analyzed during this current study are available from the corresponding author on reasonable request.
