# Supplementary material for: The Association Between Play Fighting and Information Gathering during Subsequent Contests
Source: Sci Rep. 2020 Jan 24;10:1133. doi: 10.1038/s41598-020-58063-x (PMC6981131; doi:10.1038/s41598-020-58063-x)
Supplement: Supplementary file 1 — Supporting Information. [file 41598_2020_58063_MOESM1_ESM.docx]

Supplementary Table S1 – Ethogram used to identify play fighting behaviour during video play back.

|  |  |  |
| --- | --- | --- |
| **Behaviour** | **Description** | **Reference** |
| **Play Fighting** | |  |
| *Play Fighting Invite* | Initiator piglet performs rapid face-to-face pushing actions directed at a target piglet. Pushing is often repetitive and performed in an energetic manner. Does not include deliberate/accidental shoving when the target pig is inhibiting initiator locomotion. All pushing and shoving under the udder is ignored during suckling bouts. | 1,2 |
| *Success* | Target piglet responds to the initiator piglets ‘invite’ by pushing back and engaging in a play response. Play occurs as both individuals push towards one another, with occasional head knocking and biting attempts. (See Accept Play). | 3,4 |
| *Failure* | Initiator is unsuccessful at eliciting a play response from the target individual. Target piglet either turns its head/body away from the initiator piglet, moves away without further reaction, or does not give any noticeable response to the initiator piglet’s attempts to play. (See Reject Play). | 3,4 |
| *Accept Play Fighting* | Target piglet responds to initiator piglets invite by pushing back against the head of the initiator. This results in both individuals pushing towards one another, with occasional head knocking and biting attempts. (See Success). | 3,4 |
| *Reject Play*  *Fighting* | Target piglet does not response to initiator piglet. Target piglet either turns its head/body away from the initiator piglet, moves away without further reaction, or does not give any noticeable response to the initiator piglet’s attempts to play. (See Failure). | 1,2 |
| *Third Party Interaction* | A third piglet attempts to join a pre-occurring play fight by either pushing one or both of the currently participating piglets. This interaction either results in the play behaviour finishing, one piglet being displaced from the play fight, or the continuation of the fight with a third member. | 3,4 |

1. Martin, J. E., Ison, S. H. & Baxter, E. M. The influence of neonatal environment on piglet play behaviour and post-weaning social and cognitive development. *Appl. Anim. Behav. Sci.* **163**, 69-79 (2015).

2. Brown, S. M., Klaffenböck, M., Nevison, I. M. & Lawrence, A. B. Evidence for litter differences in play behaviour in pre-weaned pigs. *Appl. Anim. Behav. Sci.* **172**, 17-25 (2015)

3. Weller, J.E., Camerlink, I., Turner, S.P., Farish, M. & Arnott, G. Socialisation and its effect on play behaviour and aggression in the domestic pig (*Sus scrofa*). *Sci. Rep.* **9***,* 4180 (2019).

4. Weller, J., Camerlink, I., Turner, S. P., Farish, M. & Arnott, G. Playful pigs: Early life play fighting experience influences later life contest dynamics. *Anim. Behav.* **In Press**

Supplementary Table S2 – Results from the two generalised linear mixed effects models used to explore the relationship between winner weight, loser weight, dyad play experience (high play vs low play), treatment (socialised vs control) and their subsequent interactions on principal component 1 (PC1) and mutual fighting duration (s).

|  | **PC1** | | | **Mutual Fighting Duration** | | |
| --- | --- | --- | --- | --- | --- | --- |
|  | χ^2^ | D.F | *p* | χ^2^ | D.F | *p* |
| Winner Weight | 0.135 | 1 | 0.714 | 0.133 | 1 | 0.716 |
| Loser Weight | 0.144 | 1 | 0.704 | 0.105 | 1 | 0.746 |
| Play Experience | 0.548 | 2 | 0.760 | 0.329 | 1 | 0.566 |
| Treatment | 4.277 | 2 | 0.118 | 3.368 | 1 | 0.066 |
| Winner Weight: Loser Weight | 1.606 | 1 | 0.205 | 0.362 | 1 | 0.547 |
| Winner Weight: Play Experience | 0.650 | 1 | 0.420 | 0.704 | 1 | 0.401 |
| Loser Weight: Play Experience | 1.382 | 1 | 0.240 | 0.019 | 1 | 0.890 |
| Winner Weight: Treatment | 3.056 | 1 | 0.080 | 1.082 | 1 | 0.298 |
| Loser Weight: Treatment | 0.489 | 1 | 0.484 | 1.725 | 1 | 0.189 |
| Play Experience: Treatment | 0.351 | 1 | 0.553 | 0.115 | 1 | 0.735 |
| Winner Weight: Loser Weight: Play Experience | 6.432 | 1 | 0.011* | 1.429 | 1 | 0.232 |
| Winner Weight: Loser Weight: Treatment | 0.617 | 1 | 0.432 | 2.088 | 1 | 0.148 |
| Winner Weight: Play Experience: Treatment | 0.054 | 1 | 0.816 | # | # | # |
| Loser Weight: Play Experience: Treatment | 2.338 | 1 | 0.126 | # | # | # |
| Winner Weight: Loser Weight: Play Experience: Treatment | 2.278 | 1 | 0.131 | # | # | # |

** indicates significance*

*# indicates than the factor/interaction was removed from the model while attempting to obtain the best fitting model*
